# Amelioration of pregnancy outcomes in a pregnant rat model with deep venous thrombosis following the transplantation of bone marrow mesenchymal stem cells

**DOI:** 10.3389/fcell.2025.1650614

**Published:** 2025-09-04

**Authors:** Yuanyuan Xie, Junrong Zhang, Rong Du, Jingjing Ji, Jingjing Lu, Haoxuan Li, Yunzhao Xu, Yuquan Zhang, Xi Cheng

**Affiliations:** ^1^ Department of Gynecology and Obstetrics, Affiliated Hospital of Nantong University, Medical School of Nantong University, Nantong, Jiangsu, China; ^2^ Research Center of Clinical Medicine, Affiliated Hospital of Nantong University, Medical School of Nantong University, Nantong, Jiangsu, China; ^3^ Department of Critical Care Medcine, Gynecology and Obstetrics Hospital of Fudan University, Yangtze River Delta Integration Demonstration Zone (QingPu), Shanghai, China

**Keywords:** pregnancy outcomes, angiogenesis, pregnancy-associated DVT, BM-MSCs, placenta

## Abstract

**Objective:**

We investigated the effects of bone marrow mesenchymal stem cells (BM-MSCs) on pregnancy outcomes in pregnant Sprague-Dawley rats with deep venous thrombosis (DVT) and explored the potential mechanisms involved.

**Methods:**

Eighteen pregnant rats were randomly divided into three groups: sham, DVT and BM-MSCs. The BM-MSCs were transfected with lentivirus carrying luciferase and cell membrane staining reagent CM-Dil to analyze the location and survival of BM-MSCs *in vivo*. We also compared the weight and length of the thrombus, the embryo absorption rate, the complete blood count, coagulation function, and D-dimer concentration of pregnant rats between the groups. Thereafter, placental blood flow was monitored by Doppler ultrasound and the number of placental blood vessels was determined by CD31 staining 7 days following BM-MSC transplantation. Finally, the expression of placental growth factor (PlGF), vascular endothelial growth factor A (VEGFA), soluble fms-like tyrosine kinase 1 (sFlt1), VEGF receptor 2 (VEGFR2) both in mRNA and protein levels were detected.

**Results:**

Reduced thrombus and improved pregnancy outcomes were observed in the BM-MSCs group. Furthermore, BM-MSCs survived and migrated to the lungs, liver, spleen, and thrombotic tissues, rather than the placenta. Doppler ultrasound indicated insufficient placental perfusion in the DVT group, which was reversed by the transplantation of BM-MSCs. BM-MSCs promoted placental angiogenesis by upregulating VEGFA and VEGFR2, and by reducing sFlt1 protein levels in the placenta.

**Conclusion:**

Our analysis suggested that BM-MSCs improve pregnancy outcomes associated with obstetric DVT by alleviating placental hypoperfusion and regulating the balance of placental pro-/anti-angiogenic factors.

## 1 Introduction

Deep venous thrombosis (DVT) is a common disease of the circulatory system and refers to the abnormal coagulation of blood in the deep veins due to a variety of factors. If a thrombus detaches and travels in the bloodstream to obstruct the pulmonary artery or its branches, this condition may result in life-threatening pulmonary embolism (PE). DVT and PE together constitute venous thromboembolism (VTE), a serious vascular disorder. Pregnant women have a 4–5-fold higher incidence of VTE when compared to non-pregnant women of the same age due to a range of key factors, including an increased blood volume, physiological hypercoagulability, and compression of the pelvic vessels by the enlarged uterus (Wang X. et al., 2025). A previous publication reported that the overall incidence of VTE during pregnancy ranges from 0.6/1000 to 1.8/1000, with a incidence of 1.0/1000 to 1.3/1000 for DVT and an incidence of 0.2/1000 to 0.4/1000 for PE (Meng et al., 2014). The occurrence of DVT in pregnant women is associated with various obstetric complications, such as recurrent pregnancy loss, intrauterine growth restriction, gestational hypertension with proteinuria, and premature separation of the placenta. PE caused by DVT is a major cause of maternal death, posing a significant economic burden on society and creating a serious threaten for the health and lives of both the mother and the fetus (Skuterud Wik et al., 2015; Cheng J. et al., 2022). The management of VTE during pregnancy presents unique challenges due to the specific characteristics of the maternal population, the need to balance maternal and fetal safety, and the limited data on anticoagulant use in this population (Wang Y. et al., 2025). Therefore, identifying appropriate treatments to improve pregnancy outcomes related to DVT during pregnancy is of great significance.

There are numerous reports on the use of mesenchymal stem cells (MSCs) to improve pregnancy outcomes. Previous research established that the use of human endometrial MSCs significantly improved pregnancy rate and litter size in a mouse model of Asherman’s syndrome (Domnina et al., 2018). Shahgaldi et al. reported that bone marrow-derived MSCs (BM-MSCs) partially corrected the imbalance of angiogenic factors observed in the CBA/J × DBA/2 mouse model of miscarriage, reducing the miscarriage rate and improving the intrauterine environment, thus providing significant benefit for fetal development (Shahgaldi et al., 2022). Moreover, therapeutic interventions utilizing MSCs have demonstrated efficacy in mitigating pregnancy-related complications through multiple mechanisms. These include amelioration of preeclampsia manifestations, modulation of immune responses, promotion of vascular development, and suppression of inflammatory processes, collectively contributing to improved gestational outcomes, and antioxidant effects (Suvakov et al., 2020).

Our group previously established a DVT model in pregnant Sprague-Dawley (SD) rats using the “stenosis” method and then administered BM-MSCs to this model. We found that MSCs promoted angiogenesis of the thrombus and reduced embryonic resorption rates, thus providing evidence for the application of MSCs in pregnancy-related VTE (Cheng X. et al., 2022). However, the mechanisms by which MSCs improve pregnancy outcomes in the pregnant rat model of DVT has yet to be fully investigated. Therefore, based on our previous work, this present study aimed to investigate: 1) the survival and localization of BM-MSCs in the pregnant rat model of DVT; 2) the effects of BM-MSCs on thrombosis, coagulation function, and pregnancy outcomes; 3) the mechanisms by which BM-MSCs improve pregnancy outcomes; and 4) the safety of BM-MSC treatment. Our aim was to provide a basis for the application of MSCs in the treatment of pregnancy-related VTE.

## 2 Materials and methods

**FIGURE 1:**
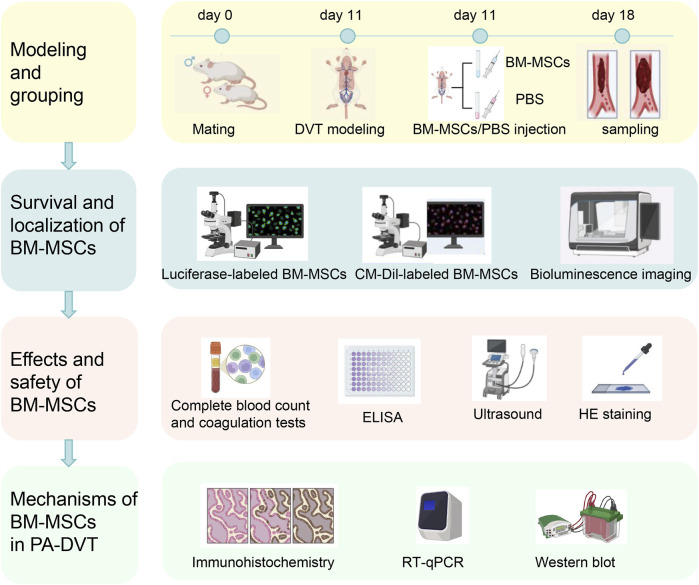
Schematic diagram of the experimental design and procedure. The first day after the detection of a vaginal plug following mating between female and male rat was designated as day 1. On day 11, deep venous thrombosis (DVT) and sham surgery models were established. Six hours after modeling, BM-MSCs (DVT + BM-MSCs group) or PBS (sham group and DVT group) were injected via the tail vein. Samples were collected 7 days later (first row). BM-MSCs were labeled with luciferase-carrying lentivirus and CM-Dil. The survival and homing of BM-MSCs were observed at 0.5 h, 24 h, 72 h, 5 days, and 7 days after transplantation through biofluorescence imaging and immunofluorescence (second row). The weight and length of the thrombus and their ratio were calculated. The total number of live-born sucking rats, embryo resorption rate, birth weight of sucking rat, placental weight, placental weight/birth weight of sucking rat, and birth weight of naturally delivered offspring (fetal weight) were measured. Complete blood count and coagulation tests were examined in the sham, DVT, and DVT + BM-MSCs groups. D-dimer concentration was detected via ELISA. Umbilical cord blood perfusion was monitored using ultrasound. Histopathological changes in the heart, liver, spleen, lungs, and kidneys tissues of pregnant rats and postpartum rats were assessed via HE staining to evaluate the safety of BM-MSCs transplantation (third row). Placental angiogenesis was assessed through CD31 immunohistochemistry. The transcriptional and protein expression levels of placental VEGFA, PlGF, sFlt1, and VEGFR2 were detected using RT-qPCR and Western blotting (fourth row).

Schematic diagram of the experimental design and procedure in this study are outlined in Figure 1.

### 2.1 Animals and experimental design

Sexually mature, nulliparous 10-week-old Sprague-Dawley (SD) female (250–300 g) and male (250–300 g) rats, including 40 females and 20 males, along with 10 three-day-old suckling rats, were procured from the Experimental Animal Center of Nantong University (Nantong, China). The experimental animals were maintained in a controlled barrier facility at the institution, where they received sterilized feed (Co60-irradiated) and purified water without restriction. Environmental conditions were strictly regulated to maintain a 12-h photoperiod and ambient temperature of 23 °C ± 3 °C. Sterilized wood chip bedding was provided and refreshed on a weekly basis or more frequently as needed to ensure optimal hygiene standards. Female and male rats were paired in a 2:1 ratio each day at 7 p.m., with the presence of a vaginal plug marking the first day of pregnancy (E0.5). Pregnant rats were then housed separately. Their diet, coat condition, excretions, and activity were closely monitored, with any abnormalities promptly addressed. Noise levels were minimized, and gentle handling was ensured to reduce stress. The study protocol received formal ethical clearance from the Institutional Animal Care and Use Committee at Nantong University, ensuring compliance with all relevant animal welfare guidelines and regulations. (Approval No.: S20221231-002).

### 2.2 Isolation, culture, and identification of BM-MSCs

Suckling rats were euthanized via CO_2_ asphyxiation and subsequently immersed in alcohol for 30 min, repeated three times. Following UV sterilization, femurs and tibias were dissected free of muscle fascia and transferred to a Petri dish containing phosphate-buffered saline (PBS). Cartilage was removed from both ends of the bones, and bone marrow was extracted by repeatedly pressing the cavity until it appeared white. The cell suspension underwent centrifugation at 1000 revolutions per minute for a duration of 5 min, followed by removal of the supernatant. This centrifugation procedure was performed in triplicate. Subsequently, the cellular pellet was reconstituted in 4 mL of DMEM supplemented with 10% fetal bovine serum and penicillin-streptomycin antibiotic solution. The cells were then maintained in a humidified incubator at 37 °C with 5% carbon dioxide atmosphere. Upon achieving 80%–90% monolayer confluence, subculturing was performed to propagate the cell line. Surface markers of MSCs were identified using a RAXMX-09011 kit (Cyagen, Guangzhou, China), while osteogenic and chondrogenic differentiation were assessed using Huxuc-90021 and RAXMX-90041 induction kits, respectively (Cyagen).

### 2.3 Lentiviral transfection of BM-MSCs

BM-MSCs at the third passage in the logarithmic growth phase were trypsinized, seeded into culture dishes, and incubated overnight. When cell confluence reached 70%–80%, an optimized dose of luciferase-expressing lentivirus (multiplicity of infection = 100) was added for transfection. Four days post-transfection, puromycin (2 μg/mL) was administered for stable selection, later reduced to 0.2 μg/mL. Successfully transfected cells were expanded for subsequent *in vivo* imaging.

### 2.4 Experimental groups, model establishment, and BM-MSC injection

**FIGURE 2:**
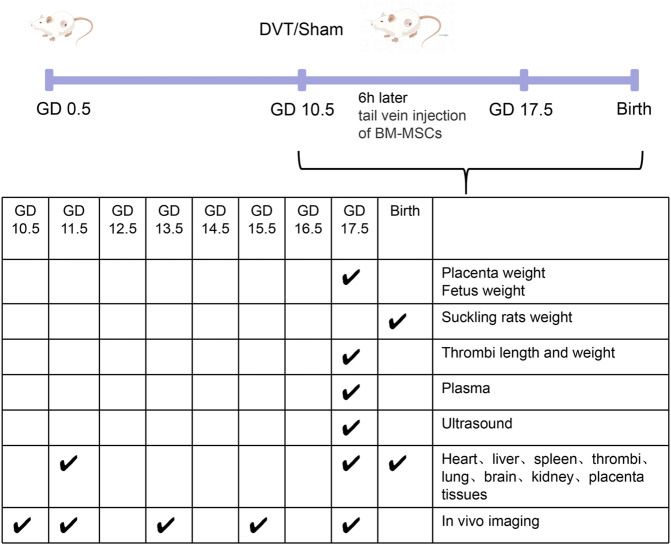
Key sampling time points and related experiments.

The experimental cohort comprised pregnant rats that were systematically randomized into three distinct groups: a sham-operated control group (n = 6), a DVT model group (n = 6), and a DVT + BM-MSC intervention group (n = 6). This study design ensured equal distribution of subjects across treatment conditions for comparative analysis. Random numbering was conducted using Arabic numerals. DVT was induced using a stenosis method, while the sham group underwent an identical procedure without ligation. In the DVT group, a 5–0 suture was placed beneath the inferior vena cava (IVC) and tied, followed by removal of a parallel 4–0 suture, leading to darkening of the proximal vessel segment, confirming model establishment. The sham group underwent blunt dissection without ligation. Six hours post-modeling, BM-MSCs were injected into the tail vein. To enhance venous dilation, tails were immersed in warm water for 3 min before disinfection. Using a visual tail vein injection system, the sham and DVT groups received 1 mL PBS, whereas the BM-MSC group received 1 mL PBS containing 2 × 10^6^ BM-MSCs. Key sampling time points and related experiments are shown in Figure 2.

### 2.5 Labeling BM-MSCs with CM-Dil

CM-Dil was dissolved in dimethyl sulfoxide to prepare a 1 μg/μL solution. BM-MSCs at the third passage in the logarithmic growth phase were resuspended in complete culture medium containing CM-Dil at a concentration of 5 μg/mL. The cellular samples underwent an initial static incubation period of 5 min under controlled conditions, maintained at 37 °C with 5% carbon dioxide concentration in a specialized cell culture incubator and then transferred to a 4 °C refrigerator for further static incubation for 15 min. Following successful cellular labeling, the samples underwent three consecutive PBS washes to eliminate any residual unbound CM-Dil dye. The labeled cells were then subjected to centrifugation at 1000 revolutions per minute for a duration of 5 min, after which the supernatant was carefully aspirated and removed.

### 2.6 Cell proliferation assay

BM-MSC proliferation was assessed using an Enhanced Cell Counting Kit-8 (CCK-8; Beyotime, Shanghai, China). Cell suspensions (2 × 10^5^ cells/mL) were seeded in 96-well plates (100 μL per well) in MSC serum-free medium. Viability was measured daily for 7 days. Before conducting the measurements, 10 μL of CCK-8 solution was introduced into every well and the plates were maintained at 37 °C for 2 h. Subsequently, absorbance readings were obtained at a wavelength of 450 nm utilizing an Epoch microplate reader (BioTek, United States).

### 2.7 Ultrasound biomicroscopy

At embryonic day 17.5 (E17.5), hemodynamic parameters of umbilical vessels were quantitatively assessed through high-resolution ultrasound biomicroscopy (Vevo 2100 imaging system, Visual Sonics Inc., Toronto, Canada). For the procedure, experimental animals were maintained under general anesthesia achieved through inhalation of isoflurane (3% for induction phase, followed by 1%–3% for maintenance of anesthesia). Color Doppler identified umbilical vessels, and pulsed Doppler sample gates were positioned over umbilical arteries. Peak systolic velocity (PSV) and end-diastolic velocity (EDV) were determined from three consecutive cardiac cycles unaffected by maternal respiration. Measurements were taken from umbilical vessels near the placenta in at least three fetuses per dam.

### 2.8 Bioluminescence imaging

BM-MSCs transduced with a luciferase-expressing lentivirus were selected for stable expression. Post-surgery, 1 × 10^6^ luciferase-expressing BM-MSCs were injected via the tail vein. Cell survival and migration in DVT pregnant rats were monitored at 0.5 h, 24 h, 72 h, 5 days, and 7 days post-transplantation. Prior to imaging, The animals received an intraperitoneal injection of D-luciferin at a dose of 150 mg/kg body weight. Subsequently, bioluminescent signals were acquired through an *In Vivo* Imaging System (IVIS). Quantitative analysis of photon flux from each tissue region was performed utilizing Living Image software for image processing and quantification. (Version 3.2, Caliper Life Sciences, Alameda, CA), following previously described protocols (Wang Y. et al., 2025).

### 2.9 *In Vivo* tracking of BM-MSCs

A total of 4 × 10^6^ BM-MSCs were fluorescently labeled using 5 μg of Cell Tracker™ CM-DiI Dye (Invitrogen, United States). The labeling procedure involved an initial incubation period of 5 min at 37 °C, immediately followed by a 15-min incubation at 4 °C. Following labeling, cells were washed twice with PBS to remove any unincorporated dye. The final cell concentration was standardized to 2 × 10^6^ cells/mL through appropriate dilution with culture medium. Labeled BM-MSCs (1 × 10^6^ cells/100 g) were intravenously administered to DVT pregnant rats 6 h post-surgery. Following a 24-h period, experimental animals were euthanized and multiple organs (including cardiac tissue, hepatic tissue, splenic tissue, pulmonary tissue, renal tissue, placental tissue, and cerebral tissue) were collected. The harvested specimens underwent fixation in 4% paraformaldehyde solution for 24 h, followed by sequential dehydration using 20% and 30% sucrose solutions. Subsequently, the biological samples were embedded in optimal cutting temperature compound (OCT) and sectioned into 8 μm thick slices for further histological analysis, stained with DAPI, and analyzed for BM-MSC distribution via fluorescence microscopy.

### 2.10 Complete blood count and coagulation tests

Blood samples (2–3 mL) were collected into sodium citrate tubes and gently inverted for proper mixing. Plasma was separated via centrifugation (1,500 rpm, 15 min, 4 °C) and transferred into microcentrifuge tubes. Complete blood counts were performed using an automated hematology analyzer, while coagulation parameters [activated partial thromboplastin time (APTT), prothrombin time (PT), thrombin time (TT) and fibrinogen (FIB)] were assessed using a coagulation analyzer (Sysmex, Japan), adhering to manufacturer protocols. Samples were processed within 2 h to ensure analytical accuracy. Final complete blood counts and coagulation parameters represent n = 6 biological replicates per group.

### 2.11 Enzyme-linked immunosorbent assay (ELISA)

The concentration of D-dimer in plasma samples was quantified employing a commercially available ELISA kit (SP13155, Spbio, Wuhan, China). Initially, 96-well microplates were coated with specific capture antibodies and maintained at 4 °C for overnight incubation. Subsequently, the plates were blocked using 5% skimmed milk solution at 37 °C for 1 hour. Following this step, both test samples and reference standards were introduced into the wells and allowed to incubate for 2 hours. The assay procedure continued with multiple washing steps, followed by sequential addition of biotin-conjugated detection antibodies, streptavidin-horseradish peroxidase conjugate, and tetramethylbenzidine (TMB) chromogenic substrate. Absorbance at 450 nm was recorded using a microplate reader, and analyte concentrations were determined via standard curve interpolation. Samples were analyzed in duplicate with appropriate controls.

### 2.12 Western blotting

Placental protein extraction was performed utilizing a commercial Total Protein Extraction Kit (Sangon Biotechnology, Shanghai, China). Following denaturation through boiling for 15 min, protein samples were resolved via SDS-PAGE and subsequently electrotransferred onto PVDF membranes. The membranes were then subjected to blocking with 5% non-fat milk at 37 °C for 2 h, followed by overnight incubation at 4 °C with specific primary antibodies targeting key angiogenic factors: PLGF (Abcam, ab196666), VEGFA (Abcam, ab46154), sFlt1 (Abcam, ab32152), and VEGFR-2 (Proteintech, 26415-1-AP). After thorough washing, membranes were exposed to HRP-linked secondary antibodies (Abcam) for 2 h at ambient temperature. Protein detection was achieved using an enhanced chemiluminescence system (Pierce, Thermo Fisher Scientific), with band visualization performed on a Tanon 2500 Gel Imaging System. Quantitative analysis of protein expression levels was conducted using ImageJ software (NIH, United States), with β-actin serving as an internal control for normalization purposes.

### 2.13 Quantitative real-time PCR (qRT-PCR)

Placental RNA extraction was performed with TRNzol Universal reagent (Beijing, China), followed by quality assessment using a NanoDrop 2000C spectrophotometer (Thermo Scientific). The extracted RNA met stringent quality criteria, exhibiting concentrations exceeding 300 ng/μL and A260/A280 ratios above 1.9. Reverse transcription was carried out with HiScript™ II Q RT SuperMix for qPCR (+ gDNA wiper) (Vazyme, Nanjing, China) to generate cDNA. Quantitative real-time PCR analysis was performed on a LightCycler 480 instrument (Roche, Basel, Switzerland) employing ChamQ Universal SYBR qPCR Master Mix (Vazyme). Gene-specific primers, designed according to GenBank sequences (detailed in Table 1), were utilized for amplification. Relative mRNA expression was calculated through the comparative threshold cycle (2^−ΔΔCt^) method.

**TABLE 1:** Primer sequences used for real-time RT-PCR.

Target gene	Forward primers, 5′–3′	Reverse primers, 5′–3′
*PLGF*	cca act cgt ccc tgc tga atg ac	gga acc gtg gct ggc ttc ttc
*VEGFA*	cgg tgt ggt ctt tcg tcc ttc tta g	agg gat ggg ttt gtc gtg ttt ctg
*Flt1*	gag cat cta tca ggc agc gga ttg	cga ccc act ctt cac acg aca ag
*VEGFR2*	ggc gat gtt agt gct ttg tgt gtt g	ttg ctc ctt cct tcc tac cag tcc

### 2.14 Hematoxylin and eosin (HE) staining

Heart, liver, spleen, lung, and kidney tissues (n = 6 per group) were paraffin-embedded following 4% formaldehyde fixation. Serial sections (5 μm) were prepared, with two sections per sample subjected to HE staining. The HE staining was performed according to the instructions of the reagent kit (Beyotime, C0105M, China).

### 2.15 Immunohistochemical staining

Paraffin sections were dewaxed, rehydrated, and treated with 3% hydrogen peroxide for 10 min. Antigen retrieval was performed using a retrieval solution (Solarbio, CHN) at 100 °C for 15 min. Sections were blocked with goat serum (37 °C, 30 min) and incubated overnight at 4 °C with CD31 antibody (Abcam, ab182981). After PBS washes, an HRP-conjugated secondary antibody (Absin, China) was applied (37 °C, 1 h). Staining was visualized using DAB (Zsbio, China). CD31 expression was quantified by calculating the number of positive vessels per unit area in five randomly selected ×400 microscopic fields.

### 2.16 Statistical analyses

The experimental data were systematically organized using Microsoft Excel and subsequently subjected to statistical analysis through GraphPad Prism software (Version 5, GraphPad Software Inc., La Jolla, CA). For comparative assessments between two experimental groups, unpaired Student's t-tests were employed, whereas one-way analysis of variance (ANOVA) was implemented for evaluating differences among multiple groups. Quantitative findings are presented as arithmetic mean ± standard error of the mean (SEM), with a predetermined threshold of *P* < 0.05 indicating statistical significance.

## 3 Results

### 3.1 Morphology, multilineage differentiation potential, and flow cytometric phenotype of BM-MSCs conformed to the basic characteristics of mesenchymal stem cells

**FIGURE 3:**
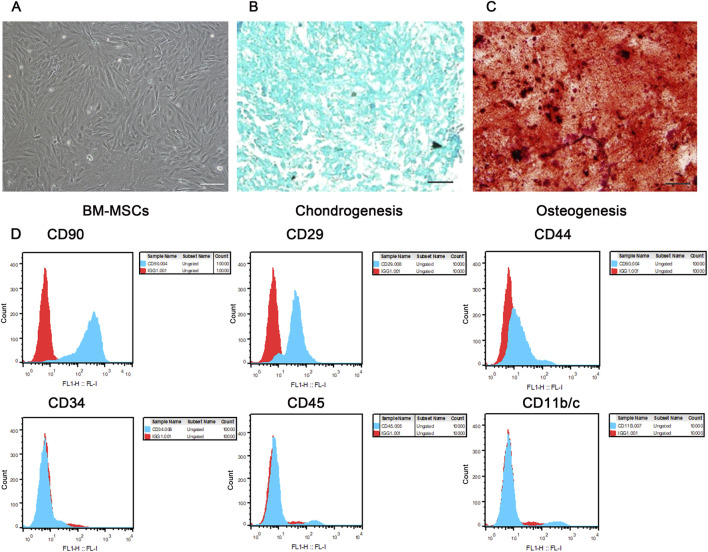
Morphology, multilineage differentiation potential, and immunophenotypic identification of BM-MSCs. **(A)** Morphology of P3 BM-MSCs, showing typical long spindle or fusiform shape with whirlpool-like growth (scale bar = 100 μm); **(B)** Alcian blue staining of P3 BM-MSCs indicating successful chondrogenic induction (scale bar = 100 μm); **(C)** Alizarin red staining of P3 BM-MSCs indicating successful osteogenic induction (scale bar = 100 μm); **(D)** Flow cytometric immunophenotypic identification of BM-MSCs. Blue histograms represent specific antibody staining and red histograms denote isotype controls, with comprehensive flow cytometry analysis confirming the characteristic mesenchymal phenotype of BM-MSCs (CD90^+^/CD29^+^/CD44^+^ and CD34^-^/CD45^-^/CD11b/c^−^) as shown in the revised high-resolution image.

As shown in Figure 3A, the extracted cells exhibited the typical spindle-shaped morphology of mesenchymal stem cells and grew in a swirling pattern. These cells were successfully induced to differentiate into cartilage (Figure 3B) and bone (Figure 3C). Flow cytometric phenotype analysis revealed that these cells were positive for CD90, CD29, and CD44, and negative for CD34, CD45, and CD11b/c (Figure 3D). These findings are consistent with the basic characteristics of MSCs as defined by the Mesenchymal and Tissue Stem Cell Committee of the International Society for Cellular Therapy.

### 3.2 BM-MSCs survived and migrated to the lung, liver, spleen, and thrombus in a pregnant rat model of DVT

**FIGURE 4:**
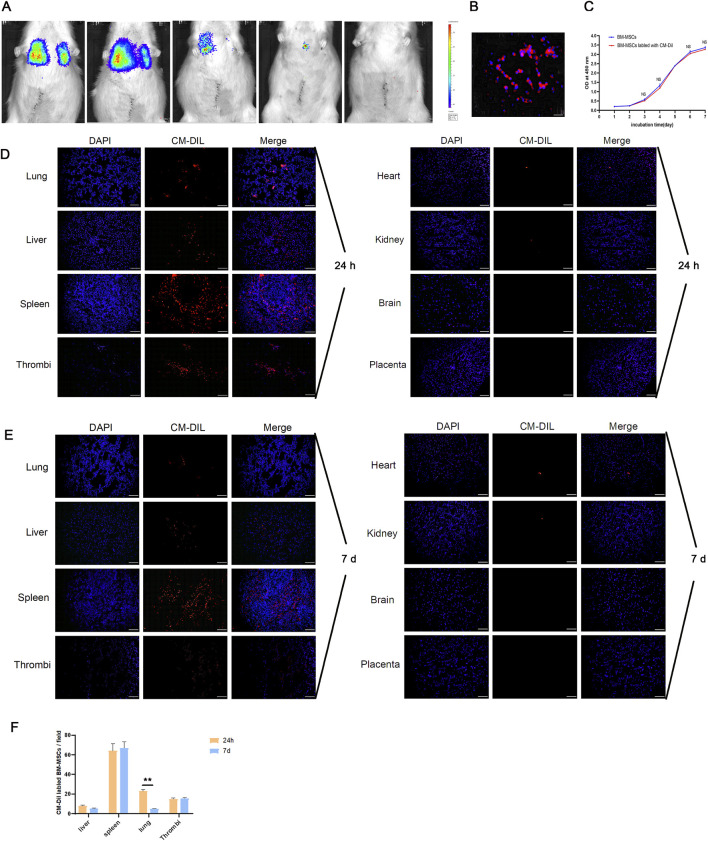
Survival and homing of BM-MSCs in the pregnant rat model of DVT. **(A)** Survival status of BM-MSCs was monitored via biofluorescence imaging at 0.5 h, 24 h, 72 h, 5 d, and 7 d. Obvious biofluorescence signals were observed in the thoracic cavity at 0.5 h and 24 h, which significantly decreased by day 3 and 5 and disappeared by day 7; **(B)** Fluorescence microscopy observation of CM-Dil-labeled BM-MSCs on slides showed obvious red fluorescence on the cell membrane (scale bar = 100 μm); **(C)** CCK-8 proliferation assay comparing the proliferation ability of CM-Dil-labeled and unlabeled BM-MSCs showed no significant difference (NS: no statistical significance); **(D)** Fluorescence microscopy observation of CM-Dil-labeled BM-MSCs localization 24 h after transplantation showed numerous red fluorescent signals in the lung, liver, spleen, and thrombus tissues, but no obvious signals in the heart, kidney, brain, and placenta (scale bar = 100 μm); **(E)** Fluorescence microscopy observation of CM-Dil-labeled BM-MSCs localization 7 days after transplantation showed red fluorescent signals in the lung, liver, spleen, and thrombus tissues, but no obvious signals in the heart, kidney, brain, and placenta (scale bar = 100 μm); **(F)** Statistical analysis of CM-Dil-positive cells in various tissues showed a significant decrease in fluorescent cells in the lung by day 7 compared to 24 h (*P* < 0.01).

The survival status of exogenous BM-MSCs *in vivo* is crucial if we are to understand their functionality and the mechanisms involved. Therefore, we used bioluminescence imaging to monitor the survival of luciferase-labeled BM-MSCs. As shown in Figure 4A, BM-MSCs survived in the pregnant rat model of DVT and predominantly accumulate in the thoracic cavity. Over time, the number of cells gradually decreased, and the fluorescent signal disappeared by day 7 post-transplantation. To further track the migration and homing of BM-MSCs, we labeled the cells with CM-Dil (Figure 4B). CCK-8 assays demonstrated that CM-Dil had no significant effect on the proliferative capacity of BM-MSCs (Figure 4C). The CM-DiI-labeled BM-MSCs were intravenously injected into pregnant rats with DVT. The homing of BM-MSCs was observed under a fluorescence microscope at 24 h and 7 days post-transplantation (Figure 4D). Analysis showed that BM-MSCs (red) colonized in the lungs, liver, spleen, and thrombus at both time points (Figures 4D,E), with no significant red fluorescent signal observed in the placenta, thus suggesting that BM-MSCs may have directly reached the thrombus site to exert their therapeutic effect and may not have reached the placental tissue. Furthermore, the number of BM-MSCs colonized in the lungs by day 7 was significantly reduced when compared to 24 h (Figure 4F). This was similar to the bioluminescence imaging results, thus indicating that the number of transplanted BM-MSCs gradually decreased over time.

### 3.3 BM-MSCs improve thrombus formation and hypercoagulability in the pregnant rat model of DVT

**FIGURE 5:**
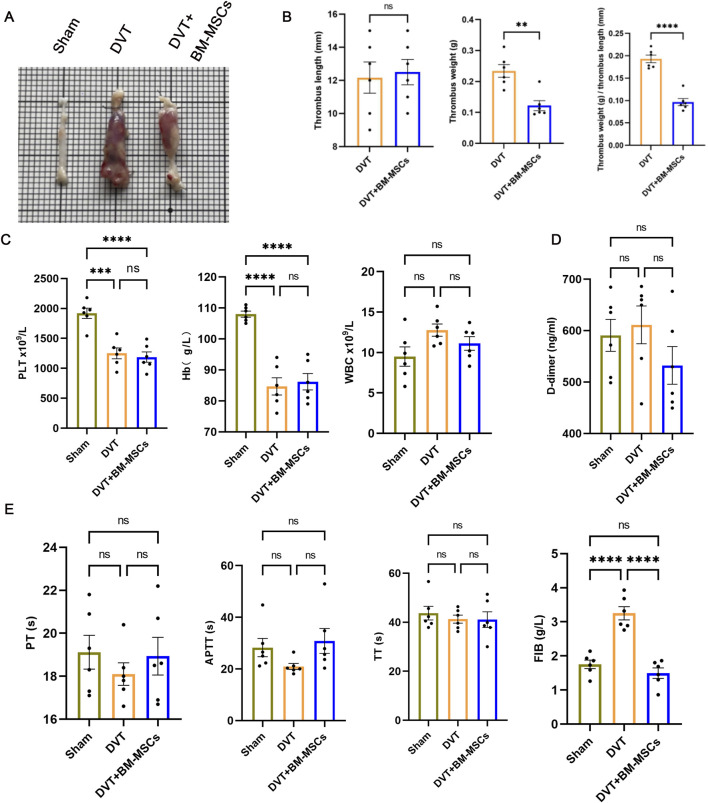
Effects of BM-MSCs transplantation on thrombus morphology and coagulation function in the pregnant rat model of DVT. **(A)** Representative anatomical images of thrombi. Compared to the sham group, obvious thrombus formation was observed in the DVT and BM-MSCs groups, with the thrombus in the BM-MSCs group being smaller than that in the DVT group; **(B)** No significant difference in thrombus length was observed between the DVT and BM-MSCs groups. Compared to the DVT group, the BM-MSCs group had smaller thrombus weight (*P* < 0.01) and thrombus weight/length ratio (*P* < 0.0001); **(C)** Compared to the sham surgery group, platelets (*P* < 0.001 and *P* < 0.0001) and hemoglobin (*P* < 0.0001, *P* < 0.0001) were significantly decreased in the DVT and BM-MSCs groups, with no significant difference in white cell count among the three groups; **(D)** No significant difference in D-dimer levels was observed among the three groups; **(E)** No significant difference in prothrombin time (PT), activated partial thromboplastin time (APTT), and thrombin time (TT) was observed among the three groups. Compared to the sham group, fibrinogen (FIB) was significantly increased in the DVT group (*P* < 0.0001), but this increase was reversed by BM-MSCs treatment (*P* < 0.0001).

The weight and length of the thrombus formed in the IVC were measured on postoperative day 7 (GD17.5), and their ratio was calculated. As shown in Figures 5A,B, when compared to the DVT model group, the BM-MSCs treatment group had a significantly lower thrombus weight and a significantly lower ratio of thrombus weight to length (Figure 5B), thus suggesting that BM-MSCs treatment improved thrombus formation in the pregnant rat model of DVT, thereby alleviating disorders of the maternal blood circulation. This was consistent with our previous findings (Cheng X. et al., 2022). Furthermore, we evaluated complete blood count, coagulation tests and D-dimer levels in pregnant rats on postoperative day 7 (GD17.5), as shown in Figures 5C–E. The specific values of the complete blood count, coagulation tests and D-dimer are shown in Table 2. The levels of platelets (PLT) and hemoglobin (Hb) in the DVT and BM-MSCs group were significantly lower than the sham group, likely due to rapid utilization of platelets during thrombus formation and activation of the coagulation cascade, as well as hemoglobin loss within a short period (Figure 5C). Statistical analysis revealed comparable PLT and Hb concentrations between subjects receiving BM-MSCs and those with DVT. Fibrinogen (FIB), a crucial coagulation factor, serves as the primary determinant of fibrin formation and exhibits pivotal involvement in venous thrombogenesis. During pregnancy, FIB exhibits a physiological increase, and rises sharply in the event of thrombus formation. As shown in Figure 5D, the level of FIB in the DVT model group was significantly higher than the sham group, thereby significantly promoting thrombus formation. Compared to the DVT group, the level of FIB was significantly lower in the BM-MSCs treatment group, thus suggesting that BM-MSCs treatment improved hypercoagulability. Furthermore, there were no significant differences in APTT, PT, TT and D-dimer levels when compared between the groups (Figures 5D,E). Collectively, these findings indicate that BM-MSC treatment mitigates thrombus burden and hypercoagulability in pregnant rats with DVT, although it does not significantly restore platelet or hemoglobin levels by day 7 post-operation.

**TABLE 2:** Results of complete blood count, coagulation tests and D-dimer concentration in the sham, DVT, and DVT+BM-MSCs groups (Mean ± Standard Error of Mean).

Indicator	Sham	DVT	DVT + BM-MSCs
PLTx10^9^/L	1919.00 ± 86.45	1250.00 ± 89.89^###^	1187.00 ± 86.86^####^
Hbx10^9^/L	108.00 ± 0.97	84.67 ± 2.78^####^	86.17 ± 2.66^####^
WBCx10^9^/L	9.50 ± 1.20	12.77 ± 0.76	11.12 ± 0.84
PT(s)	19.12 ± 0.78	18.10 ± 0.52	18.93 ± 0.88
APTT(s)	28.28 ± 3.54	20.95 ± 1.19	30.87 ± 4.83
TT(s)	43.73 ± 2.79	41.27 ± 1.65	41.08 ± 3.19
FIB(g/L)	1.75 ± 0.12	3.25 ± 0.19^####^	1.49 ± 0.15****
D-dimer(ng/mL)	590.70 ± 31.23	611.10 ± 36.73	532.20 ± 36.54

^#^ indicates statistically significant differences compared with the sham-operation group, with ^#^ *P* < 0.05, ^##^ *P* < 0.01, and ^###^ *P* < 0.001, ^####^ *P* < 0.0001. * indicates statistically significant differences compared with the DVT (deep venous thrombosis) model group, with ^****^ *P* < 0.0001.

### 3.4 BM-MSCs improved pregnancy outcomes and placental insufficiency in the pregnant rat model of DVT

**FIGURE 6:**
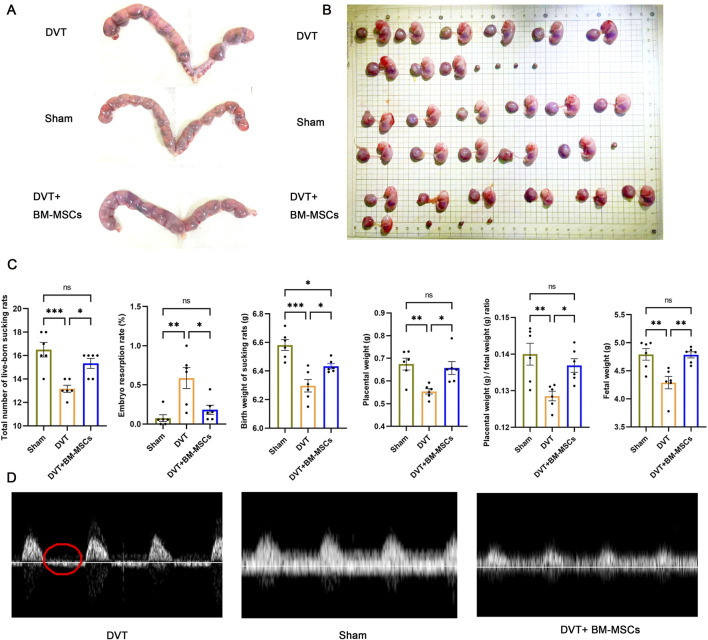
Effects of BM-MSCs transplantation on pregnancy outcomes. **(A)** Representative anatomical images of uterine horns in the sham, DVT, and BM-MSCs groups; **(B)** Representative anatomical images of placentas and embryos in the three groups; **(C)** Compared to the sham group, the DVT group showed significant decreases in total number of live-born sucking rats (*P* < 0.001), birth weight of sucking rat (*P* < 0.001), placental weight (*P* < 0.01), placental weight/birth weight of sucking rat (*P* < 0.01), and fetal weight (*P* < 0.01), with an increase in embryo resorption rate (*P* < 0.01). Compared to the sham group, no significant statistical difference was observed except for a slight decrease in birth weight of sucking rat (*P* < 0.05) in the BM-MSCs group. Compared to the DVT group, the BM-MSCs group showed significant increases in total number of live-born sucking rats (*P* < 0.05), birth weight of sucking rat (*P* < 0.05), placental weight (*P* < 0.05), placental weight/birth weight of sucking rat (*P* < 0.05), and fetal weight (*P* < 0.05), with a decrease in embryo resorption rate (*P* < 0.01). **(D)** Color atlas of umbilical vessels and doppler flow spectrum waveform of the umbilical artery. Compared to the sham group, the DVT model group exhibited absent or reversed end-diastolic flow. The part circled in red is the end-diastolic flow waveform.

As shown in Figures 6A–C, the embryo resorption rate was significantly higher in the DVT group than in the sham group. Furthermore, the average embryo and placenta weights were also significantly lower. Compared to the DVT group, the transplantation of BM-MSCs significantly reduced the embryo resorption rate and significantly increased the average embryo and placenta weights. Next, we analyzed the number and birth weight of newborn rats in the three groups. We found that the BM-MSCs treatment group had a higher number of live pups (Figure 6C), thus suggesting that BM-MSCs treatment improved pregnancy outcomes. Adequate placental blood circulation is the most basic condition for ensuring normal fetal development. To further explore the factors underlying the improvement of adverse pregnancy outcomes in pregnant DVT rats by BM-MSCs, we assessed umbilical artery and vein blood flow using small animal ultrasound on G17.5 (7 days post-modeling). By analyzing the blood flow spectrum waveform in the umbilical artery, we found that when compared to the sham group, the DVT model group exhibited absent or reversed end-diastolic flow (Figure 6D), thus indicating placental insufficiency. Surprisingly, BM-MSCs transplantation did not result in absent or reversed end-diastolic flow, thus indicating that BM-MSCs transplantation significantly improved pregnancy outcomes and placental insufficiency in the pregnant rat model of DVT.

### 3.5 BM-MSCs promoted placental angiogenesis by regulating the balance of pro-angiogenic/anti-angiogenic factors in the placenta

**FIGURE 7:**
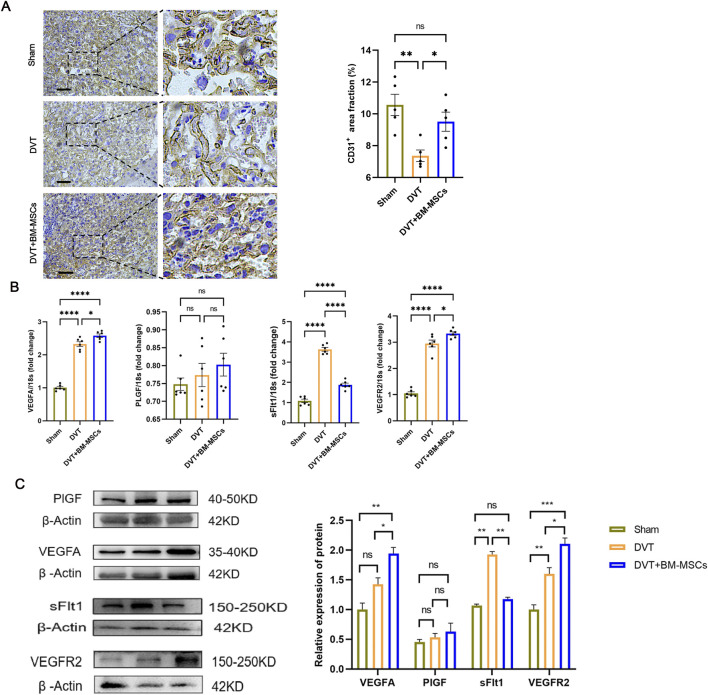
Effects of BM-MSCs transplantation on placental angiogenesis and angiogenesis-related genes. **(A)** CD31 immunohistochemical staining of placental tissue (scale bar = 200 μm). The brown ring-like luminal structures represent CD31-positive placental microvessels. Compared with the DVT group, the area proportion of placental microvessels in the BM-MSCs group was significantly higher (*P* < 0.05). **(B)** The mRNA expression of PlGF, VEGFA, sFlt1, and VEGFR2 in the placental tissues. Compared with the sham group, the mRNA levels of VEGFA (*P* < 0.0001), sFlt1 (*P* < 0.0001), and VEGFR2 (*P* < 0.0001) were significantly increased in the DVT group, while there was no significant statistical difference in PlGF mRNA levels. Compared with the DVT group, the mRNA levels of VEGFA (*P* < 0.05) and VEGFR2 (*P* < 0.05) were significantly increased in the BM-MSCs group, while the mRNA level of sFlt1 was significantly decreased (*P* < 0.0001). There was also an increase in PlGF mRNA levels, but the difference was not statistically significant. **(C)** The protein expression of PlGF, VEGFA, sFlt1, and VEGFR2 in the placental tissues. Compared with the sham group, there was no significant statistical difference in the protein levels of VEGFA and PlGF in the DVT group,and the level of sFlt1 was decreased (*P* < 0.05), but the level of VEGFR2 was significantly increased (*P* < 0.01). Compared with the DVT group, the protein levels of VEGFA (*P* < 0.05) and VEGFR2 (*P* < 0.05) were significantly increased in the BM-MSCs group, while the protein level of sFlt1 was significantly decreased (*P* < 0.05). There was no statistical significance in the protein level of PlGF between the DVT and BM-MSCs group.

To investigate the mechanism by which BM-MSCs improve placental perfusion, we next performed immunohistochemical staining for CD31 in placental tissues (Figure 7A). The brown ring-like luminal structures represent CD31-positive placental microvessels. Statistical analysis revealed that the placental microvessels in the BM-MSCs group were significantly denser than those in the DVT model group, thus suggesting that BM-MSCs promoted placental angiogenesis. Subsequently, we evaluated the mRNA and protein levels of factors related to placental vascular growth and development (VEGFA, PlGF, sFlt1, and VEGFR2 in placental tissues (Figures 7B,C). The BM-MSCs treatment group exhibited markedly elevated expression of VEGFA and its cognate receptor VEGFR2 at both transcriptional and translational levels when compared to the DVT group, concomitant with a substantial decrease in the anti-angiogenic mediator sFlt1. Notably, PlGF expression remained comparable across all experimental groups (Figure 5C). These findings collectively indicate that BM-MSCs administration effectively modulates the equilibrium between pro-angiogenic and anti-angiogenic mediators within placental tissue.

### 3.6 Safety of BM-MSCs treatment in the pregnant rat model of DVT

**FIGURE 8:**
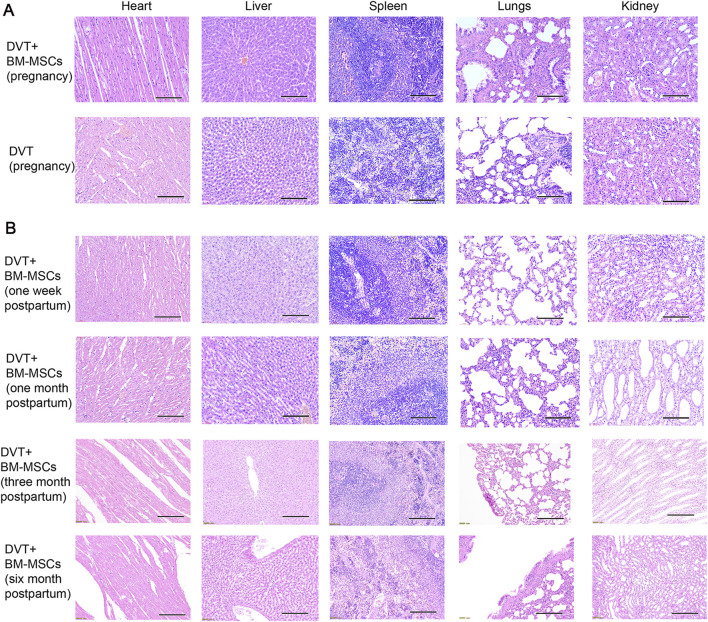
Safety of BM-MSCs treatment in the pregnant rat model of DVT. **(A)** HE staining of heart, liver, spleen, lungs and kidneys tissues of pregnant rats in the DVT group and BM-MSCs treatment group on day 18 (scale bar = 100 μm). No pathological changes such as tumors or thrombosis were observed. **(B)** HE staining of heart, liver, spleen, lungs, kidneys tissues of rats in the BM-MSCs treatment group on the 1 week, 1 month, 3 months and 6 months postpartum (scale bar = 100 μm). No pathological changes such as tumors or thrombosis were observed.

To investigate the safety of BM-MSCs during pregnancy and postpartum, we performed HE staining of the maternal heart, liver, spleen, lungs, and kidneys from the DVT and BM-MSCs group on GD17.5 (7 days post-BM-MSCs treatment), as shown in Figure 8A. No significant pathological changes, such as tumorigenesis, malformations, or thrombus formation, were observed in the vital organs of pregnant rats. With regards to the long-term safety of BM-MSCs, we did not detect any significant pathological changes in the heart, liver, spleen, lungs, and kidneys tissues of female rats in the BM-MSCs group on the 1 week, 1 month, 3 months, and 6 months postpartum (Figure 8B).

## 4 Discussion

In this study, we investigated DVT during pregnancy and used the stenosis method to establish a DVT model in pregnant rats. Based on our previous research, we found that although BM-MSCs cannot migrate to the placenta, they can improve pregnancy outcomes by enhancing placental blood perfusion in pregnant rats with DVT. This may be related to their ability to regulate the balance between placental pro-angiogenic and anti-angiogenic factors.

The function and mechanism of MSCs depends on their survival and homing *in vivo*. An MSC niche is an aggregate formed by the clustering of MSCs within tissues or on tissue engineering scaffolds. As a special microenvironment, these niches can enhance the survival and differentiation capabilities of MSCs and improve their ability to repair tissues. The formation of an MSC niche involves multiple factors, including cell adhesion molecules, cytokines, growth factors, and chemical substances, which can influence the migration and localization of MSCs. CM-Dil and GFP are often used to track the migration and homing of MSCs. CM-Dil is a lipophilic red fluorescent dye that labels cell membranes and is characterized by its lack of cytotoxicity and impact on cell proliferation. However, the fluorescence of CM-Dil may diminish with cell passages, thus affecting the final cell tracking results. GFP, a protein introduced into cells via lentiviral transfection, persists through cell passages. Although GFP is highly stable and safe, certain organs such as the hippocampus naturally emit green fluorescence, potentially leading to false-positive results. In a rat model of acute pancreatitis, Huang et al. used lentiviruses carrying luciferase to transfect placenta-derived stem cells and found that MSCs could survive after tail vein injection, with the number of viable cells decreasing over time (Huang et al., 2021). After labeling the placenta-derived stem cells with CM-Dil, we found that MSCs were distributed in the lungs, liver, pancreas, spleen, duodenum, and colon. In this study, BM-MSCs transfected with lentiviruses carrying luciferase were used to observe their survival through *in vivo* imaging and CM-Dil-labeled BM-MSCs were used to track their migration and homing. Our results revealed that MSCs primarily survived in the thoracic cavity and migrated and localized to the liver, spleen, lungs, and thrombus tissue, but not the placenta; these findings were consistent with the aforementioned study. In contrast, Wang et al. tracked human umbilical cord MSCs labeled with GFP in a preeclampsia model and found that when injected intravenously, GFP-MSCs reached the placenta and kidneys of the pregnant rats but were not distributed in the heart, lives, lungs, or kidneys of the fetal rats (Wang et al., 2016). Wu et al. reported that locally injected GFP-MSCs migrated to the placenta in a model of preeclampsia. We believe that a variety of factors may have influenced MSc tracking, including the type of model, labeling material, route and site of administration, and tracking method (Wu et al., 2020). We hypothesize that BM-MSCs transplanted through the tail vein injection may not locate to the placenta, possibly due to the obstruction of the IVC in this particular model.

VTE during pregnancy is closely linked to adverse pregnancy outcomes. Stefansk et al. reported that VTE caused by antiphospholipid antibody syndrome during pregnancy is often complicated by recurrent miscarriages (Stefanski et al., 2018). Furthermore, other studies have found that venous thrombosis during pregnancy can be complicated by conditions such as preeclampsia, placental abruption, and fetal growth restriction (Devis and Knuttinen, 2017; Da Silva Santos et al., 2017; Cheng J. et al., 2022). These conditions are driven by a common mechanism involving maternal hypoperfusion and placental circulatory disturbance (Alexander and Cleary, 2014; Ananth et al., 2018). Our research group previously constructed a DVT model in pregnant rats and detected an increased embryo resorption rate as an adverse pregnancy outcome; this rate was reduced by the transplantation of BM-MSCs. Therefore, based on our previous results, this current study further investigated the causes of adverse pregnancy outcomes in the pregnant rat model of DVT and the mechanisms by which BM-MSCs can improve pregnancy outcomes.

Doppler ultrasound is a clinically commonly used method to assess fetal status, and umbilical artery blood flow is known to be closely related to fetal growth, survival, and prognosis (Cahill et al., 2021; Zhao and Shen, 2024). The umbilical artery S/D ratio (systolic peak velocity/diastolic end velocity) can provide insights into the maternal placental blood supply (Zhao and Shen, 2024). Normally, both systolic and diastolic waveforms exist and occur in the same direction in the umbilical artery Doppler spectrum. When the diastolic end flow in the umbilical artery disappears or reverses, this is considered as a strong indication of fetal hypoxia, a critically dangerous condition that is closely associated with placental hypoperfusion (Aljunaidy et al., 2016; Rubin et al., 2021). In this study, we used the VEVO 2100 small animal ultrasound system to locate the umbilical cord blood vessels near the placenta and capture umbilical artery blood flow waveforms. Similar to the rabbit intrauterine growth restriction model constructed by Hodges et al. (Hodges et al., 2013), our pregnant rat model of DVT also exhibits placental dysfunction due to hypoperfusion, thus explaining the occurrence of adverse pregnancy outcomes in the pregnant rat model of DVT. We further found that BM-MSC treatment not only reduced thrombus weight and length, and their ratio, improved the hypercoagulable state and pregnancy outcomes, but also reversed the placental hypoperfusion state in pregnant rats with DVT, thus prompting us to explore the specific mechanisms of BM-MSCs.

BM-MSCs exhibit a definite pro-angiogenic effect, and it has been demonstrated that MSCs regulate placental angiogenesis in a number of pregnancy-related diseases. Platelet endothelial cell adhesion molecule-1 (PECAM-1, CD31) is a cell adhesion molecule expressed on the surface of hematopoietic and endothelial cells and can serve as a marker for neovascularization (Song et al., 2025). The positive expression of CD31 reflects the density of neovascular formation. By performing immunohistochemical staining for CD31 in the placenta, we found that blood vessels in the BM-MSCs treatment group were significantly more abundant than those in the DVT model group. Therefore, we further investigated the expression of angiogenesis-related factors in placenta from different groups.

Clinical cases and experimental animal studies have shown that the coordinated expression of VEGFA, PlGF, and their receptors (VEGFR1/Flt1 and VEGFR2) is crucial for normal placental vascular development, adequate trophoblast invasion, and successful pregnancy (Jena et al., 2020; Shahgaldi et al., 2022; Pérez-Gutiérrez and Ferrara, 2023). VEGFA and PlGF both belong to the VEGF family. VEGFA promotes angiogenesis, induces endothelial cell growth, reduces cell apoptosis, and increases vascular permeability while also promoting trophoblast invasion and remodelling of the spiral artery (Andraweera et al., 2012). PlGF is mainly expressed in the placenta, heart, and lungs, although its specific physiological role remains unclear (Huang et al., 2023; Paterson et al., 2024; Raja Xavier et al., 2024). Evidence suggests that under pathological conditions, PlGF plays a key role in regulating VEGF-dependent angiogenesis (Krishnan et al., 2015). Placental vascular development is predominantly divided into two stages (Mayhew et al., 2004). The initial phase is characterized by the formation of novel vascular sprouts (sprouting angiogenesis), facilitating capillary network expansion through enhanced endothelial cell proliferation and motility, coupled with the incorporation of perivascular support cells. During this stage, the expression of placental angiogenic factors is dominated by VEGFA and VEGFR2, which are provided by trophoblast cells and stromal cells. The second stage includes the extension of these new branches (non-branching angiogenesis) to produce capillaries exceeding the length of the villi, thus resulting in the twisting of blood vessels into capillary loops. These capillary loops protrude towards the syncytiotrophoblast to form the vascular-syncytial membrane, where the nuclei of syncytiotrophoblast cells are pushed aside to reduce the exchange distance between maternal and fetal circulation. During this stage, the expression of VEGFA and VEGFR2 decreases, while the expression of PlGF, Flt1, and sFlt1 increases. When the expression of these placental angiogenic factors is dysregulated, placental vascular development is impaired, thus leading to pathological pregnancy (Mayhew et al., 2004).

In addition, when anti-angiogenic factors are overproduced (such as sFlt1), they can disrupt normal placental angiogenesis and development, which is particularly important in the pathogenesis of placental circulatory disorders. As an alternative splice isoform of VEGFR1, sFlt1 is a potent angiogenic inhibitor that can sequester VEGF and PlGF from the circulation, thereby impeding their interaction with endogenous receptors (Siboto et al., 2024). In the second and third trimesters of pregnancy, the main mechanism of placental vascular expansion is non-branching angiogenesis; this ensures the elongation of terminal capillary loops, thereby maximizing nutrient exchange. This is partly mediated by PlGF, which increases endothelial cell proliferation, while an increase in sFlt-1 levels stimulates capillary loop extension and vascular sprouting by inhibiting VEGF-mediated vascular branching. The occurrence of preeclampsia is often associated with an imbalance between pro-angiogenic and anti-angiogenic factors. Compared with normotensive pregnant women, preeclamptic women exhibit upregulated levels of sFlt-1 expression in both the placenta and serum, while the serum levels of VEGFA and PlGF are reduced. Consistent evidence also indicates that these changes are observed weeks before the onset of preeclampsia symptoms (Roubalova et al., 2024). Therefore, the sFlt-1/PlGF ratio is often used as a predictive and prognostic indicator for preeclampsia. The reduction in this potent anti-angiogenic factor, sFlt-1, is biologically significant as sFlt1 functions as a soluble decoy receptor that sequesters pro-angiogenic ligands (VEGFA, PlGF). This decrease directly coordinates with our observed upregulation of VEGFA, PlGF, and VEGFR2 (Figure 7C), collectively shifting the placental angiogenic balance toward neovascularization. The observed reduction in sFlt1 following BM-MSC therapy suggests a partial restoration of the pro-/anti-angiogenic balance within the placenta. This decrease may relieve VEGF signaling inhibition, promote non-branching angiogenesis in late gestation, and facilitate the formation of terminal capillary loops, thereby improving maternal–fetal exchange. Therefore, the decrease in sFlt1 observed in our study is not merely a biochemical change but likely represents a key mechanism by which BM-MSCs ameliorate placentatal circulatory disorders in DVT-complicated pregnancies and support normal fetal development.

**FIGURE 9:**
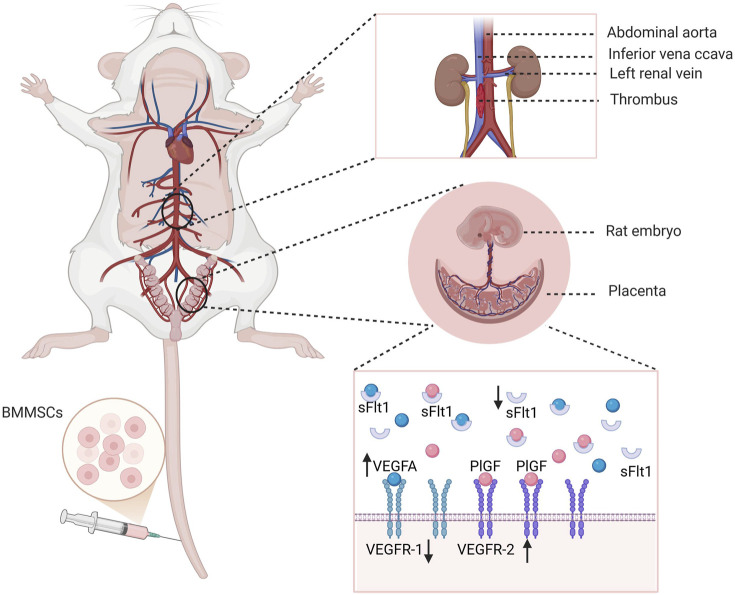
Mechanism diagram of this study. BM-MSCs may improve pregnancy outcomes in two ways. On the one hand, BM-MSCs treatment reduces thrombus weight and improves maternal blood circulation disorders, thereby alleviating placental perfusion insufficiency caused by insufficient maternal circulation perfusion. On the other hand, BM-MSCs promote placental angiogenesis by upregulating placental VEGFA and PlGF levels and reducing sFlt-1, regulating the critical balance between pro-angiogenic and anti-angiogenic factors, thereby improving placental circulation disorders and further improving pregnancy outcomes. Created with BioRender.com.

We found that the protein levels of placental VEGFA and VEGFR2 were also significantly increased, while the protein level of sFlt1 was significantly reduced after BM-MSCs treatment. In a mouse model of recurrent miscarriage constructed by Shahgaldi et al., MSC transplantation also upregulated the mRNA expression levels of placental VEGF, PlGF, and Flt1, while reducing the protein level of sFlt1, thus correcting the dysregulation of placental angiogenic factors, and improving pregnancy outcomes (Shahgaldi et al., 2022). This result is not surprising because MSCs not only produce angiogenic factors, including VEGF and PlGF, in a cell stress-dependent manner, they also directly stimulate cell expression through homing. The reduction of anti-angiogenic factor, sFlt1, promotes placental angiogenesis under pathological conditions and supports normal fetal development. Mechanism diagram of this study is outlined in Figure 9. In summary, BM-MSCs may improve pregnancy outcomes in two ways. On the one hand, after BM-MSCs treatment, maternal circulatory disorders were improved, thus alleviating placental hypoperfusion caused by insufficient maternal circulatory perfusion. On the other hand, BM-MSCs regulated the critical balance between pro-angiogenic and anti-angiogenic factors by upregulating the placental levels of VEGFA and PlGF and by reducing the levels of sFlt-1, thus promoting placental vascular formation and improving placental circulatory disorders, further improving pregnancy outcomes.

This study has several limitations that should be acknowledged. First, while we conducted *in vivo* investigations to examine the localization of - in pregnant rats with DVT and their impact on pregnancy outcomes, the cellular-level effects on placental tissues remain unexplored. Second, although we analyzed the expression of several angiogenesis-related molecules, the specific regulatory mechanisms and signaling pathways involved require more comprehensive investigation. Third, we did not establish the potential influences of varying cell dosages and transplantation frequencies on pregnancy outcomes. Forth, alternative ways of injecting the BM-MSCs into the DVT rats may take effect, specifically intra-amniotic injection. Future studies will directly investigate and compare the efficacy of intra-amniotic injection *versus* systemic delivery for BM-MSCs in this complex DVT model. These limitations highlight the need for future studies to validate our findings through cellular-level experiments, mechanistic exploration of molecular pathways, systematic evaluation of transplantation parameters, and alternative injected methods.

In summary, we found that BM-MSCs injected via the tail vein can survive in pregnant rats with DVT and can directly home to the thrombus site to exert therapeutic effects. However, BM-MSCs may not home to the placenta to directly act on placental cells. In addition, BM-MSCs can alleviate maternal blood circulation disorders by improving thrombosis in the pregnant rat model of DVT and simultaneously regulate the balance between placental pro-angiogenic and anti-angiogenic factors to promote placental angiogenesis, thus working together to increase placental perfusion and further improve pregnancy outcomes. No significant morphological changes were observed in the vital organs of the mothers following the transplantation of BM-MSCs. This study provides a novel and safe therapeutic strategy for treating adverse pregnancy outcomes associated with deep venous thrombosis during pregnancy and offers new insights into the mechanisms by which BM-MSCs improve pregnancy outcomes.

## Data Availability

The raw data supporting the conclusions of this article will be made available by the authors, without undue reservation.
